# Loss-of-function variants affecting the STAGA complex component *SUPT7L* cause a developmental disorder with generalized lipodystrophy

**DOI:** 10.1007/s00439-024-02669-y

**Published:** 2024-04-09

**Authors:** Johannes Kopp, Leonard A. Koch, Hristiana Lyubenova, Oliver Küchler, Manuel Holtgrewe, Andranik Ivanov, Christele Dubourg, Erika Launay, Sebastian Brachs, Stefan Mundlos, Nadja Ehmke, Dominik Seelow, Mélanie Fradin, Uwe Kornak, Björn Fischer-Zirnsak

**Affiliations:** 1 Institute of Medical Genetics and Human Genetics, Charité – Universitätsmedizin Berlin, corporate member of Freie Universität Berlin and Humboldt Universität zu Berlin, 13353 Berlin, Germany; 2 Max Planck Institute for Molecular Genetics, FG Development and Disease, Berlin, Germany; 3 Institute of Chemistry and Biochemistry, Department of Biology, Chemistry and Pharmacy, Freie Universität Berlin, Berlin, Germany; 4 Exploratory Diagnostic Sciences, Berlin Institute of Health, Charité – Universitätsmedizin Berlin, Berlin, Germany; 5 Core Unit Bioinformatics (CUBI), Berlin Institute of Health, Charité – Universitätsmedizin Berlin, Berlin, Germany; 6 Service de Génétique Moléculaire et Génomique, CHU, Rennes, F-35033 France; 7 Univercity Rennes, CNRS, INSERM, IGDR, UMR 6290, ERL U1305, Rennes, F-35000 France; 8 Service de Cytogénétique et Biologie cellulaire, Hôpital Pontchaillou - CHU Rennes, 2 rue Henri Le Guilloux – Rennes cedex 9, France, Rennes, F-35033 France; 9 Department of Endocrinology and Metabolism, Charité – Universitätsmedizin Berlin, corporate member of Freie Universität Berlin and Humboldt-Universität zu Berlin, 10117 Berlin, Germany; 10 German Centre for Cardiovascular Research, partner site Berlin, Berlin, Germany; 11 Berlin Institute of Health, Charité – Universitätsmedizin Berlin, Berlin, Germany; 12 Service de Génétique Clinique, Centre Référence Déficiences Intellectuelles CRDI, Hôpital Sud - CHU Rennes, 16 boulevard de Bulgarie - BP 90347, Rennes cedex 2, Rennes, F-35203 France; 13 Service de Génétique, CH Saint Brieuc, St Brieuc, 22000 France; 14 Institute of Human Genetics, University Medical Center Göttingen, Göttingen, Germany

**Keywords:** Lipodystrophy, Wiedemann-Rautenstrauch syndrome, *SUPT7L*, Aberrant splicing, Progeroid disorder, STAGA complex

## Abstract

Generalized lipodystrophy is a feature of various hereditary disorders, often leading to a progeroid appearance. In the present study we identified a missense and a frameshift variant in a compound heterozygous state in *SUPT7L* in a boy with intrauterine growth retardation, generalized lipodystrophy, and additional progeroid features. *SUPT7L* encodes a component of the transcriptional coactivator complex STAGA. By transcriptome sequencing, we showed the predicted missense variant to cause aberrant splicing, leading to exon truncation and thereby to a complete absence of SUPT7L in dermal fibroblasts. In addition, we found altered expression of genes encoding DNA repair pathway components. This pathway was further investigated and an increased rate of DNA damage was detected in proband-derived fibroblasts and genome-edited HeLa cells. Finally, we performed transient overexpression of wildtype SUPT7L in both cellular systems, which normalizes the number of DNA damage events. Our findings suggest *SUPT7L* as a novel disease gene and underline the link between genome instability and progeroid phenotypes.

**Supplementary Information:**

The online version contains supplementary material available at 10.1007/s00439-024-02669-y.

## Introduction

Lipodystrophy, the partial or generalized loss of adipose tissue, is a clinical sign in several rare monogenic disorders and is often leading to a progeroid phenotype (Nolis 2014; Jéru 2021). The molecular basis of these conditions is heterogeneous and ranges from alterations of the extracellular matrix to processes regulating DNA repair and transcription (Jéru 2021). The best known disease with progeroid features and a systemic lipodystrophy is Hutchinson-Gilford Progeria syndrome (HGPS; MIM: 176670) due to aberrant splicing of pre-lamin A causing alterations of the nuclear envelope (Eriksson et al. 2003; De Sandre-Giovannoli et al. 2003). Other conditions like Werner syndrome (WRN; MIM: 277700) and the group of Cockayne syndrome entities (CS; MIM: 216400) affect DNA repair and transcriptional mechanisms (Henning et al. 1995; Yu et al. 1996). Another disorder with impaired transcription is Wiedemann-Rautenstrauch syndrome (WDRTS; MIM: 264090), an ultra-rare autosomal recessive neonatal progeroid syndrome. WDRTS is caused by variants in the catalytic domains of RNA polymerase 3 subunits *POLR3A* or *POLR3B*, which hinder the synthesis of small RNAs (Sepehri and Hernandez 1997; Lessel et al. 2018; Paolacci et al. 2018; Wambach et al. 2018; Wu et al. 2021). Transcriptional regulation processes are important for regulating gene activity and for processing transcripts in a cell type- and tissue-dependent manner. Mostly, these processes depend on the interplay of several multi-protein complexes performing different functions (Thomas and Chiang 2006). One such protein complex is the human SPT3-TAF-GCN5 acetylase (STAGA) complex, a nuclear localized multiprotein complex playing a role in various intracellular pathways (Zhao et al. 2008; Nagy et al. 2009; Tan et al. 2014; Hirsch et al. 2015), such as splicing, transcription factor binding (Liu et al. 2003; Zhang et al. 2014), and DNA repair (Martinez et al. 2001; Liu et al. 2008; Gamper and Roeder 2008; Gamper et al. 2009; Switonski et al. 2021).

Here we report on the identification of compound heterozygous variants in *SUPT7L*, encoding a component of the STAGA complex, in an individual with generalized lipodystrophy, cataracts and a neonatal tooth, sharing features with Wiedemann-Rautenstrauch syndrome and other progeroid disorders.

## Materials and methods

### Affected individual

Peripheral blood samples were taken from the affected individual and his parents. In addition, a skin biopsy was obtained from the affected individual and dermal fibroblasts were cultivated according to standard procedures.

### Genome sequencing

Genome sequencing was performed on the DNA samples from individuals I-1, I-2 and II-1. Libraries were prepared with the DNA tagmentation based library preparation kit (Illumina) without PCR, with 500 ng gDNA input. Library preparation was followed by clean up and/or size selection using SPRI beads (Beckman Coulter Genomics). After library quantification (Qubit, Life Technologies) and validation (Agilent Tape Station), equimolar amounts of library were pooled. The library pools were quantified using the Peqlab KAPA Library Quantification Kit and the Applied Biosystems 7900HT Sequence Detection System and then sequenced on an Illumina NovaSeq6000 sequencing instrument with a paired-end 2 × 150 bp protocol (Target coverage 300x or 1200 Gb per sample). Sequence reads were mapped to the genome version GRCh37 (UCSC hg19) with the Burrows-Wheeler Aligner (BWA MEM). Single-nucleotide variants and short indels were called with the Genome Analysis Toolkit (GATK) according to the GATK Best Practices (McKenna et al. 2010; DePristo et al. 2011). We used Jannovar (Jäger et al. 2014) for variant annotation, and Varfish for filtering and further data analysis as described previously (Holtgrewe et al. 2020).

### Sanger sequencing

Sanger sequencing was performed to validate the variants in *SUPT7L* in DNA samples from the proband and his parents. Exon 2 and 3 of *SUPT7L* were amplified using the FIREPol Mastermix (SOLIS BIODYNE, Tartu, Estonia) in a ProFlex PCR System (Thermo Fisher Scientific, Dreieich, Germany). Sequencing was performed using BigDye Terminator v3.1 Cycle Sequencing Kit (Thermo Fisher Scientific, Dreieich, Germany) and electrophoresis was carried out on an ABI 3730 DNA Analyzer (Thermo Fisher Scientific, Dreieich, Germany). All primer sequences are listed in Supplementary Table 1.

### Cell culture

Dermal fibroblasts and HeLa cell line were cultured in DMEM (4.5 g/l glucose, Gibco, Thermo Fisher Scientific, Dreieich, Germany) with 10% fetal calf serum (Gibco, Thermo Fisher Scientific, Dreieich, Germany), 1% UltraGlutamine (Lonza, Basel, Switzerland) and 1% penicillin/streptomycin (Lonza, Basel, Switzerland) at 37°C and 5% CO_2_.

### RNA extraction and cDNA synthesis

Cells were lysed in Trizol (Thermo Fisher Scientific, Dreieich, Germany) and total RNA was extracted using the Direct-Zol RNA Miniprep kit (Zymo Research, Freiburg, Germany). cDNA was transcripted using the RevertAid H Minus First Strand cDNA Synthesis Kit (Thermo Fisher Scientific, Dreieich, Germany).

### Qualitative RT-PCR

cDNAs were amplified with primers in exon 2 and 4 of *SUPT7L* using the FIREPol Mastermix (SOLIS BIODYNE, Tartu, Estonia) in a ProFlex PCR System (Thermo Fisher Scientific, Dreieich, Germany). After Agarose-Gel-Electrophoresis a gel extraction of the amplified fragments S1 of II-1 was performed using the QIAquick Gel Extraction Kit (Qiagen, Venlo, Netherlands). Sequencing of these products were performed as described above. All primer sequences are listed in Supplementary Table 1.

### Quantitative RT-PCR

Quantitative PCR was performed on cDNA samples (generated as described above) and carried out using Eva Green (Solis BioDyne, Tartu, Estonia) on a QuantStudio 03 system (Thermo Fisher Scientific, Dreieich, Germany) using two different primer pairs. All primer sequences are listed in Supplementary Table 1.

### RNA sequencing and bioinformatics

We performed a poly-A (pA) enrichment from total RNA preparations (II-1 (three technical replicates) and three unaffected controls). Libraries were prepared using the Illumina Stranded TruSeq RNA sample preparation protocol. Library preparation started with 500 ng total RNA. After poly-A selection (using poly-T oligo-attached magnetic beads), mRNA was purified and fragmented using divalent cations under elevated temperature. The RNA fragments underwent reverse transcription using random primers. This was followed by second strand cDNA synthesis with DNA Polymerase I and RNase H. After end repair and A-tailing, indexing adapters were ligated. The products were then purified and amplified (15 PCR cycles) to create the final cDNA libraries. After library validation and quantification (Agilent Tape Station), equimolar amounts of library were pooled. The pools were quantified by using the Peqlab KAPA Library Quantification Kit and the Applied Biosystems 7900HT Sequence Detection System. The pools were sequenced on an Illumina NovaSeq6000 sequencing instrument (Illumina, SanDiego, CA, USA) with a PE100 protocol. 60–70 million sequence reads were generated. RNA-Seq reads were mapped to the human genome (GRCh38.p7) with STAR version-2.7.9a (Dobin et al. 2013). Reads were assigned to genes using FeatureCounts SUBREAD version-v2.0.1 (Liao et al. 2014). For the differential expression analyses we used DESeq2 version-1.34.0 (Love et al. 2014). The gene set enrichment analysis was carried out using CERNO algorithm from R tmod package version-0.46.2 (Zyla et al. 2019). All results are listed in Supplementary Table 2.

### Immunoblot

Proteins were extracted in RIPA buffer (150 mM NaCl, 50 mM Tris, 5 mM EDTA, 1% Triton X-100, 0.25% Desoxycholate, 5% SDS) containing protease inhibitor (cOmplete, Roche, Basel, Switzerland). 20 µg of protein per lane was separated by SDS-PAGE, transferred to nitrocellulose membrane and probed with primary antibodies. Immunoblot staining was performed for SUPT7L (rabbit anti-SUPT7L; 25606-1-AP, Proteintech, Rosemont, Illinois, USA), SUPT3H (mouse anti-SPT3, #sc-101157, Santa Cruz Biotechnology, Dallas, Texas), TAF10 (rabbit anti-TAF10, ab263967, Abcam, Cambridge, UK), Lamin A/C (mouse anti-LAMIN A/C; NB100-74451, Novusbio, Centennial, Colorado, USA) and GAPDH (anti-GAPDH, #AM4300, ThermoFisher, Massachusetts, USA). Membranes were incubated with IRDye-/ HRP-conjugated secondary antibodies. Signals were detected with OdysseyFc Imaging System and densitometric quantification was performed using Image Studio (LI-COR Biosciences, Lincoln, Nebraska USA).

### Immunofluorescence

Dermal fibroblasts were grown on glass coverslips overnight. Fixation was performed for 10 min in 4% paraformaldehyde at room temperature. The cells were permeabilized using 0.4% Triton X-100 in 3% BSA in 1x PBS for 10 min at room temperature. Immunofluorescence staining was performed for SUPT7L (rabbit anti-SUPT7L; 25606-1-AP, Proteintech, Rosemont, Illinois, USA), LAMIN A/C (mouse anti-LAMIN A/C; NB100-74451, Novusbio, Centennial, Colorado, USA), γH2A.X (mouse anti-pH2A.X (Ser139)(#05-636-I, Merck, Darmstadt, Germany) and turbo-GFP (rabbit anti-turboGFP (#TA150071 Origene, Rockville, Maryland, USA) overnight in 3% BSA in 1x PBS. Secondary antibody staining was performed using anti-mouse IgG Alexa Fluor 488 (#A21202, Invitrogen Waltham, Massachusetts USA) and anti-rabbit IgG Alexa Fluor 555 (#A21572, Invitrogen, Waltham, Massachusetts USA) for 1 h in 1x PBS at room temperature. DNA was stained by DAPI and cells were mounted in Fluoromount G (Biozol, Eching, Germany). Pictures were taken using a LSM700 (Zeiss, Oberkochen, Germany). Each experiment was performed three times.

### Cloning of CRISPR/Cas9 plasmid and generation of SUPT7L knockout HeLa cells

The single guide RNA (sgRNA) targeting the third exon of human *SUPT7L* was designed using the Benchling sgRNA design tool (www.benchling.com) and was selected based on its predicted on- and off-target scores. The sgRNA was then cloned into the PX459 vector from Addgene (#62,988) as previously described (Ran et al. 2013). The final vector was transfected using jetPEI (Polyplus, Illkirch, France) into HeLa EM2-11th cells (Weidenfeld et al. 2009) and 48 h post transfection positive clone selection was initiated with 1.5 µg/ml Puromycin (Thermo Fisher Scientific, Dreieich, Germany). Puromycin-selected single cell clones were expanded and validated by Sanger sequencing. One cell clone carrying a single nucleotide deletion 4 base pairs upstream of the PAM sequence (c.226delC) was selected and further characterized. The deletion causes a frameshift and premature stop codon leading to the gene knockout (KO) (Supplementary Fig. 1).

### Cell proliferation assay

Cell proliferation was measured using a WST-1 proliferation assay (Roche, Basel, Switzerland) according to manufacturer’s instructions. In brief, sextuplicates of each cell line (1 × 10^4^ cells/well) were seeded in microtiter plates in 100 µl medium in a humidified atmosphere (37 °C, 5% CO_2_) for 24 h. Then, 10 µl/well Cell Proliferation Reagent WST-1 were added and incubated for another 2 h. Before measurement, plates were shaken for 1 min and OD/absorbance was determined at 450 nm with 690 nm as reference against blank as background control on a microtiter plate reader (Infinite 200 pro, Tecan).

### Analysis of DNA damage

For analyzing DNA damage in dermal fibroblasts (II-1 and unaffected controls) and HeLa cells (WT and SUPT7L-KO), cells were seeded on glass coverslips and handled as described above. γH2A.X (Ser139), a marker for DNA damage (Paull et al. 2000), was stained in the cells and the number of cells with more than six foci of γH2A.X in the nucleus was quantified using immunofluorescence analyses. At least 100 cells per sample were counted and the experiment was performed three times.

### Rescue of the DNA damage phenotype

Dermal fibroblasts from the affected individual, unaffected controls and HeLa cells (WT and SUPT7L-KO) were transfected with 2 µg of pCMV6-AC-GFP + SUPT7L-WT (NM_014860.3, Origene) using the Amaxa Nucleofector® 2b (Lonza, Basel, Switzerland) according to manufacturer’s instructions. Immunofluorescence staining of turboGFP and γH2X.A was performed as described above. At least 100 transfected and untransfected cells per sample were counted and the experiment was performed three times.

### Statistical analysis

Statistical analyses were performed in Prism (GraphPad Prism 8.3) using the two-way analysis of variance (ANOVA) or students t-test. Figures were designed using Inkscape.

## Results

### Clinical description

**Fig. 1:**
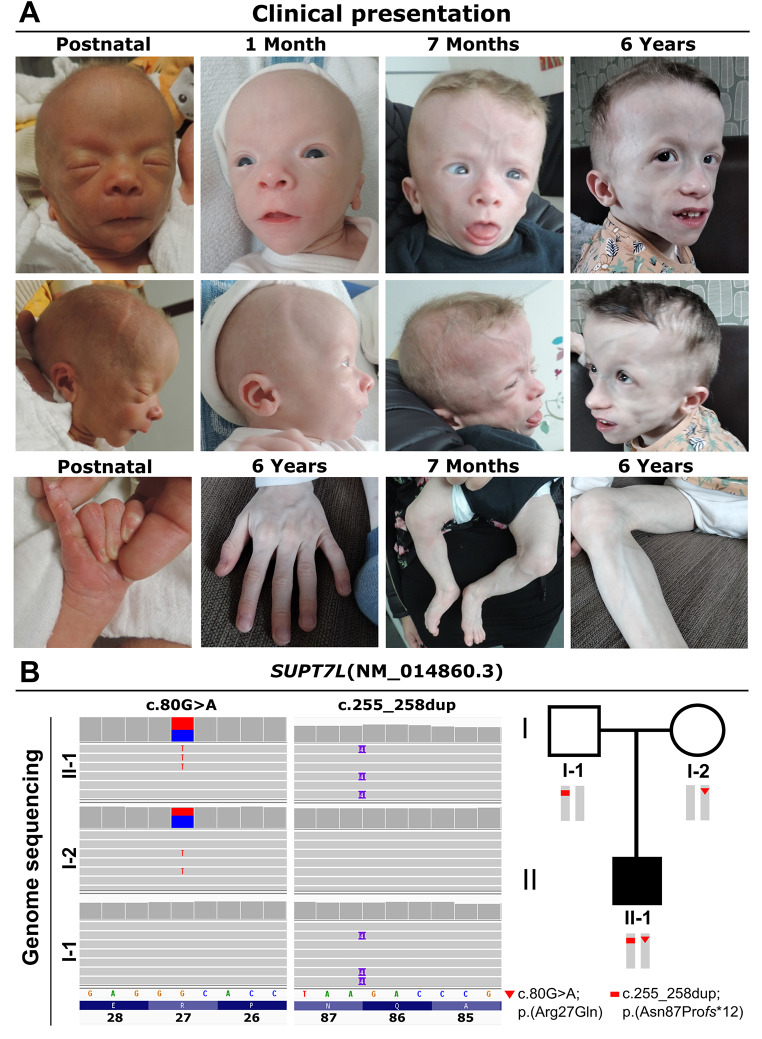
Clinical and genetic characterization of proband II-1. (**A**) Facial appearance of the affected individual II-1 postnatal, and with 1 month, 7 months, and 6 years. Note, the thin skin leading to visibility of his subcutaneous veins and generalized lipodystrophy. (**B**) We detected the variants c.80G>A and c.255_258dup in *SUPT7L* in a heterozygous state in the parents (I-1 and I-2) and in a compound heterozygous state in the index patient II-1

The affected individual II-1 is the child of healthy, unrelated parents from France. During pregnancy, intrauterine growth retardation was observed within the 3rd trimester. He was born at 36 + 4 weeks of gestation with Apgar scores 9/10/10. Birth weight was 2090 g (-2.0 SD), length was 42 cm (-3.1 SD), and head circumference (OFC) was 33 cm (-0.8 SD). Generalized lipodystrophy leading to thinned skin with visible subcutaneous veins was noted neonatally as well as a triangular face with prominent forehead, downslanting palpebral fissures, hypertelorism, deep set ears, sparse hair and eyebrows and congenital cataracts (Fig. 1A). Remarkably, he had a neonatal tooth and an inguinal hernia, which was corrected by surgery. His cerebral MRI was unremarkable, while his echocardiography showed slight pericardial effusion without cardiac malformation. At three months of age he had achieved head control but other motor developmental milestones were delayed and he started walking at four years of age.

Follow up at four years of age revealed a weight of 10.7 kg (-3.8 SD), a height of 87 cm (-4 SD) and an OFC of 49 cm (-1.7 SD). In addition, he presented with an increased muscle tone and camptodactyly of the left middle finger, which was surgically corrected. His anterior fontanel was still wide open. The last follow up at six years of age revealed a weight of 14.8 kg (-3.1 SD), a height of 96 cm (-4.4 SD) and an OFC of 49 cm (-2.4 SD). All growth parameters were continuously reduced. The OFC stagnation indicates a secondary microcephaly (Woods 2004). After loss of the neonatal tooth dentition was normal. He had a prominent abdomen with an umbilical hernia. Abdominal ultrasound revealed a progressive hepatomegaly and a renal asymmetry. He made poor eye contact, had developed no language and was in general severely developmentally delayed. Due to the intrauterine onset of the phenotype it belongs to the group of a congenital segmental progeroid disorder (Lessel and Kubisch 2019). Among them, several differential diagnoses such as Berardinelli-Seip congenital lipodystrophy (CGL1-4; MIM: 608594, 279700, 612526 and 613327), Fontaine progeroid syndrome (FPS; MIM: 612289) or Wiedemann-Rautenstrauch syndrome (WDRTS, MIM 264090) were discussed, however; none of these conditions were completely in line with the complex of symptoms observed in individual II-1 (Supplementary Table 3).

Laboratory investigations revealed normal values for creatine kinase and hepatic enzymes (ASAT, ALAT) at birth, which were elevated twofold at 10 months of age. Investigation of lipid values revealed slightly elevated total triglycerides (3.36 mmol/l; normal (*N* < 2.26 mmol/l). His cholesterol values were normal, however, his leptin levels were reduced to < 0.2 µg/l (*N* > 0.35 µg/l). He presented with hypothyroidism, which was successfully treated by L-thyroxin supplementation. His differential blood count showed hyperleukocytosis with polynuclear neutrophils and low platelet counts. At 4 years of age triglycerides were slightly elevated (2.41 mmol/l; *N* < 2.26 mmol/l). Platelets were still low at 101 × 10^9^/l (*N* > 193 × 10^9^/l). Genetic analysis by array-CGH and clinical exome sequencing revealed no pathogenic variants in known disease genes explaining this combination of symptoms.

### Disease gene identification

To decipher the genetic basis of the observed phenotype we performed trio-genome sequencing (GS) of the affected individual and his parents. Variants were evaluated using the Varfish platform (Holtgrewe et al. 2020). We assumed an autosomal recessive mode of inheritance. Initially, we tested genes associated with different forms of lipodystrophy from Genomics England PanelApp (Lipodystrophy - childhood onset (Version 4.50); ID: R158) and all candidates listed in Supplementary Table 4. to investigate whether a variant affecting a known disease gene was initially missed. We could not find any variant explaining the observed phenotype in line with the negative result from clinical exome sequencing. Next, we performed genome-wide filtering for rare homozygous or compound heterozygous variants. In addition, we tested for *de novo* variants in case the assumption of an autosomal recessive mode was incorrect. This approach revealed two heterozygous variants in *SUPT7L* (NM_014860.3).

The first variant c.80G > A is predicted to substitute an arginine at position 27 in the SUPT7L polypeptide to a glutamine. This alteration is not uniformly assessed as pathogenic by different tools: MutationTaster2 (Disease causing) (Schwarz et al. 2014), Polyphen 2 (Probably damaging) (Adzhubei et al. 2013) and CADD score: 32 (Kircher et al. 2014). It was only found in a heterozygous state in gnomAD (version v4.0.0) (https://gnomad.broadinstitute.org/) in ten individuals from different ancestry groups (MAF: 0.000006196). Besides the predicted amino acid change, this variant might form a cryptic splice acceptor site according to VarSeak (Supplementary Fig. 2A). MobiDetails reported the highest propensity for abnormal splicing for adipose tissue (AbSplice max. tissue score 0.04) (Baux et al. 2021; Wagner et al. 2023). We found this alteration to be inherited from the clinically unaffected mother (I-2) (Fig. 1B).

The second variant c.255_258dup was detected in a heterozygous state and leads to a frameshift and a premature termination codon (PTC) after 12 additional codons p.(Asn87Profs*12). This paternally inherited variant was only found in a heterozygous state in gnomAD (version v4.0.0)(MAF: 0.00001573) and was predicted to be probably damaging by MutationTaster (Probably damaging)(Fig. 1B).

Both detected variants are extremely rare and have never been observed in a homozygous state. A closer inspection of the *SUPT7L* locus in gnomAD revealed an absence of homozygous loss-of-function (LoF) variants (Karczewski et al. 2020). In addition, the observed number of single nucleotide variants slightly differs from the expected number, however, the loss-of-function observed/expected upper bound fraction (LOEUF) value is 0.618, indicating that this gene is not sensitive for haploinsufficiency. This is in line with the heterozygous parents showing no signs of the disease. Neither through platforms such as GeneMatcher (Sobreira et al. 2015) nor personal communication with cooperation partners we were able to identify any other individual with homozygous or compound heterozygous variants in *SUPT7L*.

### Functional consequences of the nucleotide substitutions

**Fig. 2:**
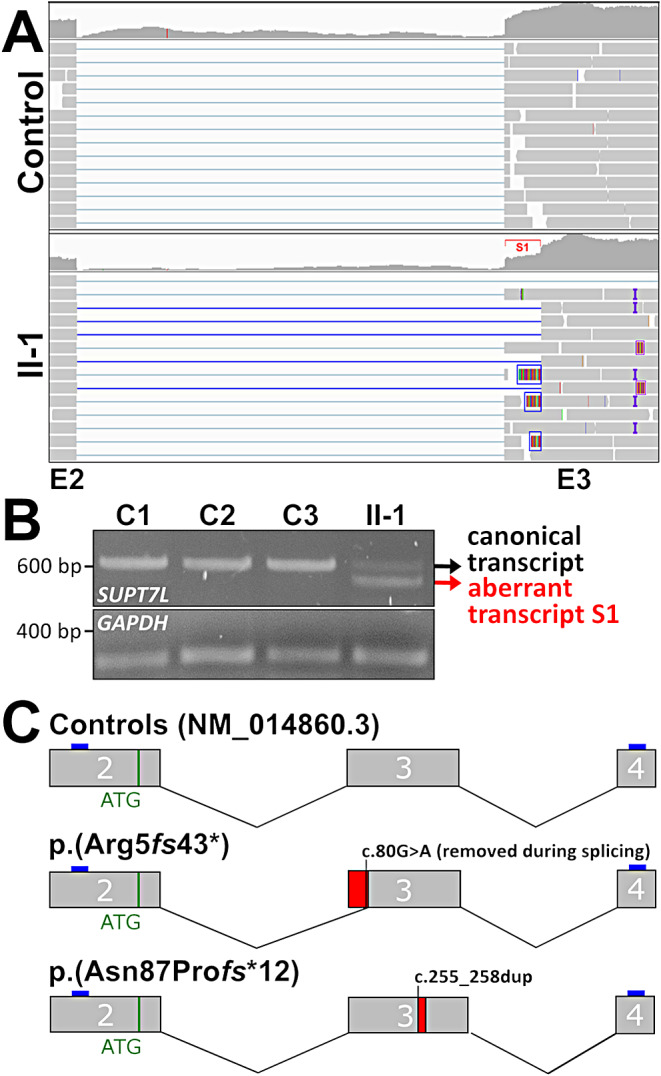
Consequences of the *SUPT7L* variants on mRNA level. (**A**) Transcriptome sequencing data from the proband’s fibroblasts and a control. IGV alignment representing exon 2–3 of *SUPT7L*. In II-1 the duplication c.255_258dup is visible in approximately 50% of the reads (purple marks and boxes). The heterozygous variant c.80G>A was not detectable in any read, however split reads fusing exon 2 with a position within exon 3 (blue lines) indicating another *SUPT7L* isoform (S1) due to aberrant splicing. In addition, clipped reads showing sequence belonging to exon 2 were detectable (Blue boxes) (**B**) RT-PCR of *SUPT7L* exon 2 to 4 resulted in two products. The upper band represents the canonical splice product while the lower band confirmed the aberrant splice product S1 (in red) in II-1. (**C**) Schematic illustration of *SUPT7L* exons 2–4 with positions of the causative variants and the resulting aberrant splicing. Primer positions used for RT-PCR (in C) are indicated as blue boxes. Red boxes indicate alterations of *SUPT7L* mRNA compared to control

In parallel to trio-GS, we performed poly A-enriched transcriptome sequencing of three technical replicates from the affected individual’s dermal fibroblasts and four matched controls. We found the variant c.255_258dup in approximately half of the sequence reads while the variant c.80G>A was not found in the transcriptome data. A closer inspection of the position c.80 in Integrative Genomics Viewer (IGV) (Robinson et al. 2011), revealed split and clipped reads directly connecting the end of exon 2 with a region within exon 3 of *SUPT7L*, indicating aberrant splicing at this position so that the alteration at position c.80 is intronic and spliced out (Fig. 2A, Supplementary Fig. 2B and 2C). Using RT-PCR and direct sequencing of the obtained PCR products after gel extraction we found both transcript variants to be stable (Fig. 2B, Supplementary Fig. 2D). In addition, using qRT-PCR and the analysis of *SUPT7L* gene expression in the transcriptome data revealed on average no strong difference in comparison to controls (Supplementary Fig. 3A and 3B). The aberrant transcript generated by exon truncation lacks 67 nucleotides (NM_014860.3:r.14_82del) and is predicted to result in a frameshift p.(Arg5*fs*43*). Of note, also using this technique, the alteration c.80G>A was not detectable on cDNA from dermal fibroblasts. These data show that both compound heterozygous *SUPT7L* variants detected in the affected individual lead to a frameshift and thereby very likely cause a complete loss of function (Fig. 2C).

### SUPT7L variants lead to loss of function and affect other components of the STAGA complex

*SUPT7L* encodes a component of the core structural module of the STAGA complex (Martinez et al. 2001), a nuclear multifunctional protein complex that plays a role in various cellular processes, such as transcription factor binding, protein acetylation and splicing (Supplementary Fig. 4) (Soutoglou et al. 2005).

**Fig. 3:**
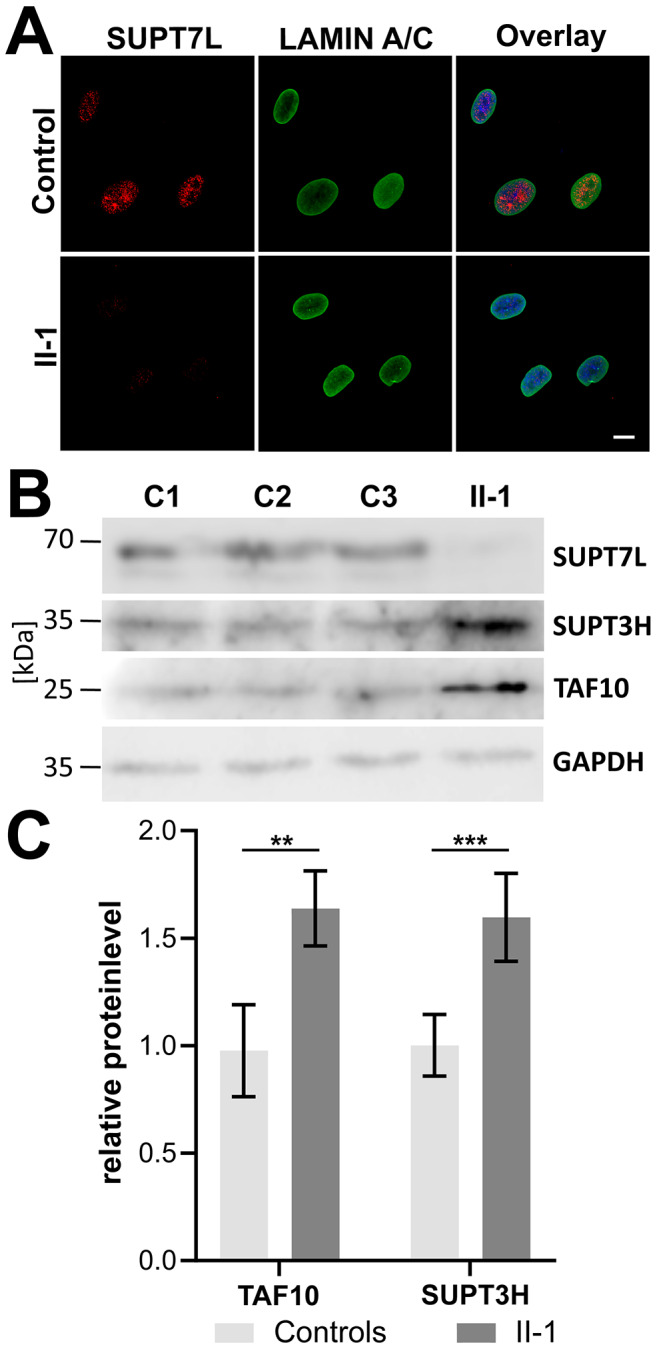
Consequences of the *SUPT7L* variants on protein level. (**A**) Immunofluorescence staining of SUPT7L in the nucleus. Representative images of SUPT7L (red) and nuclear envelope marker LAMIN A/C (green) in fibroblasts from individual II-1 and unaffected controls. SUPT7L is absent in II-1 cells. Scale: 20 μm. (**B**) Immunoblot detection of the STAGA complex components SUPT7L, SUPT3H and TAF10 in lysates of fibroblasts from individual II-1 and unaffected controls. Note the loss of the SUPT7L protein in the proband. Increased protein levels of the STAGA complex components SUPT3H and TAF10 were detected. (**C**) Quantification of immunoblot analysis for STAGA components SUPT3H and TAF10. Students T-test: P**<0.01; P***<0.001

To further investigate the pathogenicity of the observed variants we performed immunolabeling in fixed dermal fibroblasts from unaffected individuals and found SUPT7L to be localized within the nucleus. In the proband’s fibroblasts no signal was detectable indicating complete absence of the SUPT7L protein (Fig. 3A). In addition, we investigated the nuclear morphology in the proband’s fibroblasts in comparison to controls. Using immunofluorescence staining of the nuclear envelope marker LAMIN A/C, no nuclear morphology changes were observed (Fig. 3A). To further investigate the impact of the *SUPT7L* variants we performed immunoblot analyses of SUPT7L and exemplary STAGA complex components. In the cells from the affected individual, SUPT7L was absent and the protein level of SUPT3H and TAF10 was strongly increased (Fig. 3B and C).

### Loss of SUPT7L causes gene expression changes related to nuclear function and DNA damage

**Fig. 4:**
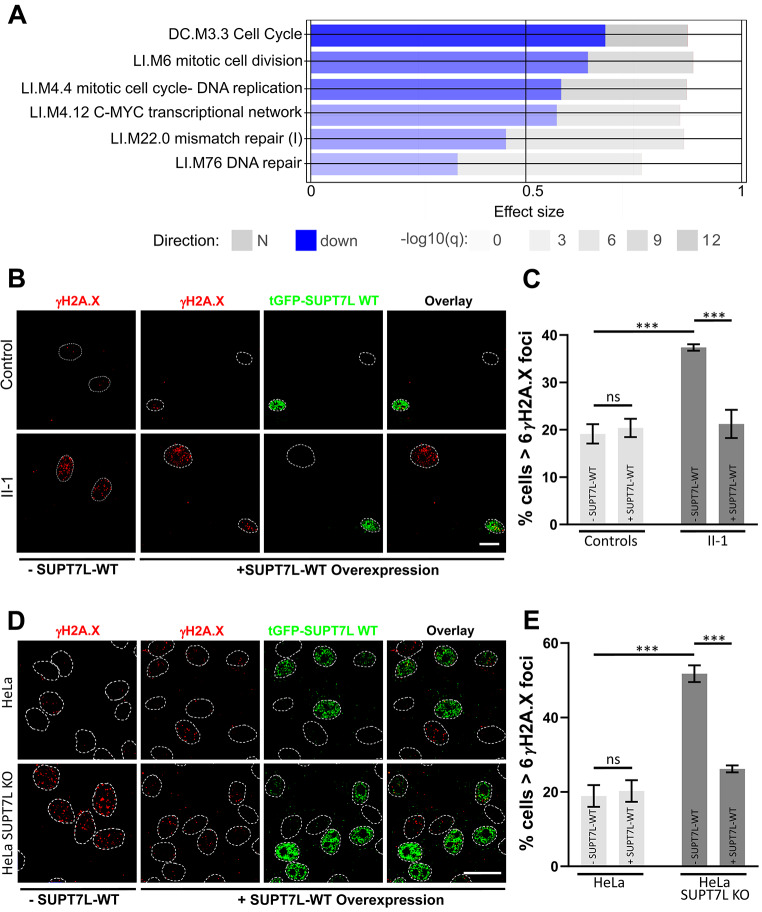
SUPT7L loss of function causes gene expression changes and leads to an increased rate of DNA damage events. (**A**) Transcriptome analysis of fibroblasts from individual II-1 compared to unaffected controls. Shown are selected deregulated gene sets affecting DNA repair, cell cycle and transcription. Downregulated genes are shown in blue, not aberrant regulated genes in grey. The effect size represents the area under the curve, the brightness of the boxes shows the significance. (**B**) Representative images of immunofluorescent staining of DNA damage marker γH2A.X (red) in fibroblasts from unaffected controls (B top) and proband II-1 (B bottom) under normal condition (-SUPT7L-WT) and transient overexpression of the turboGFP (tGFP) SUPT7L-WT fusion protein (+ SUPT7L-WT). Nuclei are labeled by white lines. (**C**) We quantified the percentage of cells with more than six γH2A.X foci under normal conditions and after transient overexpression analyzed in B. Scale bar 20 μm. Two-way ANOVA was performed; ns: not significant; P***<0.0001. (**D**) Representative images of immunofluorescent staining of DNA damage marker γH2A.X (red) in HeLa-WT (D top) and HeLa SUPT7L-KO (D bottom) under normal condition (-SUPT7L-WT) and transient overexpression of the turboGFP (tGFP) SUPT7L-WT fusion protein (+ SUPT7L-WT). Nuclei are labeled by white lines. (**E**) We quantified the percentage of cells with more than six γH2A.X foci under normal conditions and after transient overexpression analyzed in D. Scale bar 20 μm. Two-way ANOVA was performed; ns: not significant; P***<0.0001

To gain further insight into the cellular consequence of SUPT7L loss of function, we analyzed the transcriptome data of individual II-1 and four unaffected controls. Geneset enrichment analysis revealed a downregulation of several gene sets associated with DNA replication, DNA repair, cell cycle and transcription (Fig. 4A, Supplementary Figs. 5–8, Supplementary Table 2).

Due to the lack of a second proband, we generated *SUPT7L* KO HeLa cells using CRISPR/Cas9 to verify our findings in a model system (Weidenfeld et al. 2009; Ran et al. 2013) (Supplementary Fig. 1). First, we wanted to know whether the proband-derived and the genome-edited cells show altered proliferation. To analyze cell proliferation, we used the WST-1 cell proliferation assay which showed a slight reduction of proliferation rate; however, this was not significant (Supplementary Fig. 9A and 9B).

According to expression data and because the human STAGA complex is a chromatin-acetylating transcriptional coactivator associated with DNA damage binding factors (Martinez et al. 2001; Liu et al. 2008; Gamper and Roeder 2008; Gamper et al. 2009), we investigated DNA damage levels. In both cell models, we quantified the number of DNA damage events through immunofluorescence staining using a γH2A.X antibody (Paull et al. 2000). Approximately 19% of control fibroblasts showed more than six γH2A.X foci compared to 37% of the proband’s cells corresponding to an increase of 1.9-fold (Fig. 4B and C, Supplementary Fig. 10A). In addition, we also found an accumulation of DNA damage in the genome edited HeLa cells. Whereas 19% of the WT HeLa cells had over six γH2A.X foci, in the SUPT7L-KO line about 51% of the cells were above this threshold corresponding to a 2.7-fold increase (Fig. 4D and E, Supplementary Fig. 10B). Therefore, the SUPT7L KO model recapitulates the increased rate of DNA damage in the proband fibroblasts and corroborates that the loss of SUPT7L leads to problems with DNA repair.

### Overexpression of wild-type SUPT7L corrects elevated DNA damage

Next, we investigated whether a complementation with SUPT7L-WT would rescue this cellular phenotype. We transfected a plasmid carrying the wild type cDNA of SUPT7L in frame to a turboGFP into the proband’s fibroblasts and cells from unaffected controls, as well as in the SUPT7L-KO and -WT HeLa cells. 48 h post transfection, no changes were observed in the control cells, whereas the percentage of cells with more than six γH2A.X foci was reduced by approximately 95% in the proband’s fibroblasts (Fig. 4B and C, Supplementary Fig. 10C). A similar effect was seen in the SUPT7L-KO HeLa cells where the rescue effect was 81% (Fig. 4D and E, Supplementary Fig. 10C). There was no significant difference between the transfected proband’s fibroblasts and non-transfected control cells. Thus, heterologous overexpression of SUPT7L-WT completely rescued the cellular phenotype of both II-1 fibroblast and HeLa SUPT7L-KO cells.

## Discussion

In the present study we describe an individual with systemic lipodystrophy leading to a progeroid appearance and severe developmental delay resembling WDRTS in many aspects. The cardinal features of WDRTS are IUGR and a postnatal failure to thrive. The affected individuals often show a dysmorphic facial gestalt including a triangular face with a prominent forehead, widely spaced eyes with downslanting palpebral fissures and deeply set ears. Further characteristics of WDRTS include hypotrichosis with sparse scalp hair, eyebrows and eyelashes, congenital cataracts, delayed closure of the large fontanelle, a neonatal tooth, a prominent abdomen, long fingers, a prominent venous network and translucent skin due to a systemic lipodystrophy. Most of these features were also present in the here described individual, supporting the initial clinical diagnosis of WDRTS. Endocrinological and metabolic changes can also occur in WDRTS, but are more characteristic for Berardinelli-Seip congenital lipodystrophy conditions. There is also significant overlap with features of FPS in the herein described affected individual, although craniosynostosis was absent. However, no variants in the genes associated with the discussed differential diagnoses were detected neither by ES nor GS. Interestingly, the phenotypic presentation of progeroid disorders is not always suggestive for a specific genetic defect (Schnabel et al. 2021). In several cases, the diagnosis made after detection of the molecular defect differs from the initial clinical diagnosis. Many individuals with WDRTS carry causative variants in *POLR3A and POLR3B*, however, also pathogenic variants in *PYCR1*, *COL1A1* and other disease genes can cause overlapping conditions leading to the clinical diagnosis of WDRTS (Dimopoulou et al. 2013; Lessel et al. 2018; Paolacci et al. 2018; Wambach et al. 2018). In the affected individual described here, no pathogenic variants in the known disease genes associated with lipodystrophy or progeroid disorders were identified. Therefore, our finding adds *SUPT7L* as a novel candidate gene for progeroid conditions.

Both *SUPT7L* variants lead to the loss of the protein. Beside a clear frameshift variant, a predicted missense variant was found to be actually leading to a frameshift by exon truncation due to aberrant splicing. In the literature varying numbers are given how many missense or nonsense variants cause alternative splicing. However, it is estimated that this might affect at least 2% of missense or nonsense variants (Dufner-Almeida et al. 2019; Haque et al. 2024). The finding that a predicted missense variant causes aberrant splicing and converts a potentially stable but functionally altered product into a clear LoF is of importance for variant interpretation in general. However, aberrant splicing can also cause the generation of a stable isoform due to the formation of a cryptic splice site modulating disease severity (Kornak et al. 2022). In light of this work, one might speculate that the aberrant splicing induced by the *SUPT7L* variant c.80G>A might show tissue-specific differences. Indeed, AbSplice predicted the highest usage of the newly formed splice acceptor site in fat tissue, which is well in line with lipodystrophy phenotype (Wagner et al. 2023). Tissue with less usage of this splice site could produce the p.(Arg27Gln) isoform, which might be an explanation for the survival of the affected individual. This shows on the one hand that for all variants a potential effect on splicing should always be considered during the variant interpretation process. On the other hand, the identification of tissue specific impacts of cryptic splice sites can be an explanation for phenotypic variability.

The identified variants led to a complete loss of function of *SUPT7L* encoding a component of the STAGA complex. We found the protein absent in fibroblasts and SUPT7L-KO HeLa cells using immunolabeling and immunoblots, which also demonstrates the specificity of the antibody. In addition, the absence of SUPT7L leads to a compensatory upregulation of other components of the STAGA complex in the proband’s fibroblasts, but not in SUPT7L-KO HeLa cells. This is in line with previous studies showing that the depletion of SUPT7L does not cause such an upregulation of other complex components as found in the proband´s dermal fibroblasts (Liu et al. 2008). This difference between fibroblasts and HeLa cells might be due to the intrinsically high MYC expression in HeLa cells, probably due to an HPV insertion, or just because of the generally aberrant character of HeLa cells (Shen et al. 2017). We currently think that a loss of SUPT7L results in an impairment of the STAGA complex integrity and activity, but further research is necessary to fully understand the consequences of the described variants.

Transcriptome sequencing of the proband-derived fibroblasts revealed strong alterations of gene sets encoding proteins related to DNA repair, cell cycle and the MYC-related transcriptional network. In addition, the STAGA complex components TAF5L and TAF6L have also been described to interact with the c-myc network (Seruggia et al. 2019). MYC is a sequence-specific transcription factor (proto-oncogene) regulating about 15% of human genes and influences chromatin structure via the recruitment of histone acetyltransferase complexes (Yamada et al. 2004; Dang et al. 2006). The recruitment of the STAGA components TRRAP and GCN5 occurs via the direct interaction of the N-terminal activation/transformation domain of MYC with the STAGA complex (Liu et al. 2003). This leads to hyperacetylation at specific MYC-dependent genes causing an increased expression. Therefore, and according to the presented data, we speculate that loss of SUPT7L leads to a dysfunction of the STAGA complex and thereby to alteration of MYC-dependent gene regulation in fibroblasts. Although this effect may vary in other cell types, potentially due to an abovementioned variable presence of a hypomorphic SUPT7L isoform, the consequence of MYC misregulation can be more mitotic errors (Topham et al. 2015). This might also be an explanation for the fact that not more individuals with this kind of defect have been identified so far. A stronger downregulation of genes from the affected pathways is unlikely to be compatible with life. Furthermore, the strong downregulation of genes related to DNA repair are is in line with the known association of the human STAGA complex associated with DNA damage binding factors on protein level (Martinez et al. 2001; Liu et al. 2008; Gamper and Roeder 2008; Gamper et al. 2009). Accordingly, we found significantly more DNA damage in the cells from the affected individual and in the genome-edited HeLa cells. The fact that this was correctable by the complementation with wild type SUPT7L underlines the causality of both variants and a direct involvement of this complex in DNA damage recognition.

In conclusion, we present the phenotype of an individual with a severe multisystem progeroid condition caused by pathogenic *SUPT7L* variants. We suggest SUPT7L as a candidate gene for conditions with systemic lipodystrophy. In addition, these data imply SUPT7L and the STAGA complex to be important for the regulation of DNA damage control and shows that this gene is important for normal development in humans.

### Electronic supplementary material

Below is the link to the electronic supplementary material.

Supplementary Material 1

Supplementary Material 2

## Data Availability

No datasets were generated or analysed during the current study.
